# T cell specific deletion of Casitas B lineage lymphoma-b reduces atherosclerosis, but increases plaque T cell infiltration and systemic T cell activation

**DOI:** 10.3389/fimmu.2024.1297893

**Published:** 2024-03-04

**Authors:** Winnie G. Vos, Bram W. van Os, Myrthe den Toom, Linda Beckers, Cindy P.A.A. van Roomen, Claudia M. van Tiel, Bhopal C. Mohapatra, Hamid Band, Katrin Nitz, Christian Weber, Dorothee Atzler, Menno P.J. de Winther, Laura A. Bosmans, Esther Lutgens, Tom T.P. Seijkens

**Affiliations:** ^1^ Department of Medical Biochemistry, Amsterdam University Medical Centers (UMC) Location University of Amsterdam, Amsterdam, Netherlands; ^2^ Amsterdam Cardiovascular Sciences, Atherosclerosis & Ischemic Syndromes, Amsterdam, Netherlands; ^3^ Amsterdam Immunity and Infection, Inflammatory Diseases, Amsterdam, Netherlands; ^4^ Department of Genetics, Cell Biology and Anatomy, College of Medicine, University of Nebraska Medical Center, Omaha, NE, United States; ^5^ Eppley Institute for Research in Cancer and Fred & Pamela Buffett Cancer Center, University of Nebraska Medical Center, Omaha, NE, United States; ^6^ Institute for Cardiovascular Prevention (IPEK), Ludwig-Maximilians-Universität, Munich, Germany; ^7^ German Centre for Cardiovascular Research (DZHK), partner site Munich Heart Alliance, Munich, Germany; ^8^ Department of Cardiovascular Medicine and Immunology, Mayo Clinic, Rochester, MN, United States; ^9^ Munich Cluster for Systems Neurology (SyNergy), Munich, Germany; ^10^ Department of Biochemistry, Cardiovascular Research Institute Maastricht (CARIM), Maastricht University, Maastricht, Netherlands; ^11^ Walther Straub Institute of Parmacology and Toxicology, Ludwig-Maximilians-Universität München, Munich, Germany; ^12^ Department of Medical Oncology, Netherlands Cancer Institute, Amsterdam, Netherlands

**Keywords:** CBL-B, T cells, inflammation, exhaustion, atherosclerosis

## Abstract

**Introduction:**

Atherosclerosis is a lipid-driven inflammatory disease of the arterial wall, and the underlying cause of the majority of cardiovascular diseases. Recent advances in high-parametric immunophenotyping of immune cells indicate that T cells constitute the major leukocyte population in the atherosclerotic plaque. The E3 ubiquitin ligase Casitas B-lymphoma proto-oncogene-B (CBL-B) is a critical intracellular regulator that sets the threshold for T cell activation, making CBL-B a potential therapeutic target to modulate inflammation in atherosclerosis. We previously demonstrated that complete knock-out of CBL-B aggravated atherosclerosis in *Apoe^-/-^* mice, which was attributed to increased macrophage recruitment and increased CD8^+^ T cell activation in the plaque.

**Methods:**

To further study the T cell specific role of CBL-B in atherosclerosis, *Apoe^-/-^* CD4^cre^ *Cblb* ^fl/fl^ (Cbl-b^cKO^) mice and *Apoe^-/-^CD4^WT^* Cblb^fl/fl^ littermates (Cbl-b^fl/fl^) were fed a high cholesterol diet for ten weeks.

**Results:**

Cbl-b^cKO^ mice had smaller atherosclerotic lesions in the aortic arch and root compared to Cbl-b^fl/fl^, and a substantial increase in CD3^+^ T cells in the plaque. Collagen content in the plaque was decreased, while other plaque characteristics including plaque necrotic core, macrophage content, and smooth muscle cell content, remained unchanged. Mice lacking T cell CBL-B had a 1.4-fold increase in CD8^+^ T cells and a 1.8-fold increase in regulatory T cells in the spleen. Splenic CD4^+^ and CD8^+^ T cells had increased expression of C-X-C Motif Chemokine Receptor 3 (CXCR3) and interferon-γ (IFN-γ), indicating a T helper 1 (Th1)-like/effector CD8^+^ T cell-like phenotype.

**Conclusion:**

In conclusion, Cbl-b^cKO^ mice have reduced atherosclerosis but show increased T cell accumulation in the plaque accompanied by systemic T cell activation.

## 1 Introduction

Atherosclerosis is a dyslipidemia driven inflammatory disease of the arterial wall and the underlying cause of most cardiovascular disease (1). Atherosclerosis has long been recognized as an inflammatory disease (2) and clinical trials including CANTOS, COLCOT, and LoDoCo1 and 2 have fueled the search for additional anti-inflammatory therapies for atherosclerosis (3–6). Recent advances in high-parametric immunophenotyping of immune cells indicate that T cells constitute the major leukocyte population in the atherosclerotic plaque, with 25-38% of the leukocytes in the plaque being CD3^+^ T cells (7–10). Experimental studies show that T cells are involved in both the initiation and progression of atherosclerosis (11, 12). Naive CD4^+^ T cells recognize antigens, for example apolipoprotein B_100_, presented on MHC-II antigen presenting cells in the draining lymph nodes, leading to their differentiation into distinct T helper (Th) subsets, including the pro-atherogenic Th1 cells or the anti-atherogenic regulatory T cells (Tregs) (13, 14). Th1 cells migrate to the plaque where they secrete cytokines, such as TNF-α and IFN-γ, thereby contributing to plaque progression (15, 16). Tregs are considered anti-atherogenic as they suppress Th1 cells and macrophages, by production of the anti-inflammatory cytokines TGF-β and IL-10 (17–19). In advanced atherosclerosis, cytotoxic CD8^+^ T cells contribute to increased plaque vulnerability by inducing apoptosis of endothelial cells and vascular smooth muscle cells, and through granzyme B and perforin secretion. Moreover, CD8^+^ T cells regulate monopoiesis, thereby increasing circulating monocyte levels, which in turn can contribute to plaque burden (20). However, CD8^+^ T cells also have the capability to lyse pro-inflammatory T cells, including Th1, suggesting a dual role of cytotoxic T cells in atherosclerosis. Inhibiting the atherogenic effect of Th1 cells (21, 22), or enhancing Treg function (23), are potential therapeutic strategies. For example, inhibiting T cell activation by vaccination against T cell epitopes of native ApoB1 reduces atherosclerosis and vascular inflammation in mice (24). Since the role of T cells in atherosclerosis is so diverse, there is a need to explore additional T cell modulating therapeutic strategies.

Casitas B-lymphoma proto-oncogene-B (CBL-B) is an E3 ubiquitin ligase and is a critical intracellular regulator that hampers T cell activation (25, 26). After initiation of T cell activation by T cell receptor (TCR) engagement and CD28-mediated co-stimulation, CBL-B is degraded by several downstream proteins, including Phospholipase (PLC)γ and protein kinase C (PKC)θ (25), which releases the ‘natural brake’ on T cell activation. CBL-B functions as the gatekeeper of T cell activation, and as a result, CBL-B deficient T cells do not require CD28 co-stimulation for proliferation and IL-2 production, leading to increased susceptibility to auto-immunity in CBL-B^-/-^ mice (25, 26). Furthermore, CBL-B deficient T cells are less susceptible to Treg mediated suppression and anergic signals, leading to prolonged activation (27, 28).

In human atherosclerotic plaques, CBL-B is expressed in both macrophages and T cells and decreases during the progression of atherosclerosis (29). We previously demonstrated that deficiency of CBL-B aggravated atherosclerosis in *Apoe^-/-^* mice (29). Plaques of *Apoe* ^-/-^ *Cblb* ^-/-^ mice displayed increased CD8^+^ T cell infiltration and fewer CD68^+^ macrophages. While the CD8^+^ T cells from *Apoe* ^-/-^ *Cblb* ^-/-^ mice promoted monocyte/macrophage activation and recruitment to the plaque, plaque macrophages were prone to apoptosis, resulting in larger necrotic cores and, thereby, more advanced plaques. The number of splenic CD8^+^ T cells was increased and displayed a more activated phenotype, characterized by increased production of IFN-γ and granzyme B, and the CD8^+^ T cells showed resistance to Treg suppression. By limiting both T cell and monocyte/macrophage activation and recruitment, CBL-B has proven to play an important role in the progression of experimental atherosclerosis. However, the role of T cell versus macrophage specific CBL-B signaling in atherosclerosis remains unexplored. To detail the T cell specific role of CBL-B in atherosclerosis, we generated *Apoe^-/-^* mice deficient in T cell CBL-B and evaluated its effects on atherosclerosis progression and immune cell activation.

## 2 Materials and methods

### 2.1 Animals

*Cbl-b* ^fl/fl^ on a C57BL/6J background mice have been previously described (30). *Cbl-b* ^fl/fl^ mice were backcrossed to *Apoe* ^-/-^ *Cd4^cre^* mice to generate *Apoe* ^-/-^ *Cd4* ^cre^ *Cblb* ^fl/fl^ mice. The genotype of the mice was determined using tail DNA PCR with primers specified in **Supplementary Table 1**. Pups were born at a Mendelian ratio. Female *Apoe* ^-/-^ *Cd4^cre^Cblb* ^fl/fl^ (Cbl-b^cKO^) mice and *Apoe* ^-/-^ *Cd4* ^WT^ *Cblb* ^fl/fl^ littermates (Cbl-b^fl/fl^) (6-8 weeks old) were bred and housed in groups within the Animal Research Institute Amsterdam (ARIA) and were fed a 0.15% cholesterol diet (C1000 modification, Altromin Spezialfutter) ad libitum for 10 weeks. Mice were sacrificed by CO_2_ asphyxiation followed by exsanguination via cardiac puncture and subsequent harvesting of the organs. Researchers were blinded during the experiment and during the analysis. All animal experiments were performed after approval by the Committee of Animal Welfare of the University of Amsterdam (AVD1180020171666).

### 2.2 Histology

Hearts and the arterial tree were fixed overnight in 4% and 1% paraformaldehyde, respectively, and subsequently embedded in paraffin. Aortic root sections and longitudinal sections of the aortic arch and main branch points were cut in 4 µm sections, stained with haematoxylin and eosin (H&E, Merck and VWR International), and analyzed for plaque size and necrotic core size. For phenotypic parameters, immunohistochemistry for T cells (anti-CD3 1:100, AbD Serotec), CD8^+^ T cells (anti-CD8, 1:100, eBioscience), FoxP3^+^ Tregs (anti-FoxP3, 1:100, eBioscience), macrophages (Mac3, 1:100, BD Pharmingen), or α-smooth muscle actin (anti-α-SMA, 1:3000, Sigma Aldrich) was performed, and counterstained with hematoxylin. Collagen was visualized by picro Sirius Red staining (Sigma Aldrich). Plaque phenotyping was performed according to the criteria generated by Virmani, and classified as either intimal xanthoma (IX), pathological intima thickening (PIT), or fibrous cap atheroma (FCA) (31).

### 2.3 Flow cytometry

Blood was obtained by cardiac puncture and collected into tubes containing ethylenediaminetetraacetic acid (EDTA). Bone marrow was flushed from the femur using phosphate-buffered saline (PBS). Spleen, lymph nodes, and thymus were homogenized and filtered through a 70 µm cell strainer (Corning). Blood, spleen, and bone marrow were subjected to red blood cell lysis (150 mM ammonium chloride and 10 mM sodium bicarbonate, 5mM EDTA, pH 7.4). Aortas were digested for 30 minutes at 37°C using an enzyme mixture containing collagenase I (3.6 mg/ml, C0130, Sigma Aldrich), collagenase type XI (0.1 mg/ml, C7657, Sigma Aldrich), hyaluronidase (0.15 mg/ml, H3506, Sigma Aldrich), and DNase I (60 U/ml, Thermo Fisher) in RPMI medium and filtered through a 70 µm cell strainer. Cells were stained in cell staining buffer (PBS, 0,5% BSA, 5 mM EDTA) containing fluorescently labelled antibodies, for 30 minutes at 4°C. As an Fc receptor block, anti-CD16/anti-CD32 (1:1000, BioLegend, #101330) was added to samples. Prior to analysis, 7-AAD (1:1000, Thermo Fisher Scientific, #A1310) or DAPI (1:1000 (Violet laser)-1:10.000 (UV laser), ThermoFisher scientific, D21490) was added to exclude dead cells.

For intracellular staining, cells were washed with PBS and subsequently stained for 30 minutes at 4°C with Fixable Near-IR Live/Dead (1:1000 in PBS, Thermo Fisher Scientific) or Fixable Blue Dead Cell stain kit (1:1000 in PBS, Thermo Fisher Scientific). Cells were stained in cell staining buffer containing fluorescently labelled antibodies for 30 minutes at 4°C. Next, cells were fixed and permeabilized using the Foxp3/Transcription Factor Staining Buffer Set (ThermoFisher Scientific) according to the manufacturers protocol. Cells were stained in permeabilization buffer containing intracellular fluorescently labelled antibodies for 30 minutes at 4°C.

Stained cells were analyzed on a LSRFortessa Cell Analyzer (BD Biosciences), Symphony A1 Cell Analyzer (BD Bioscience), or BD FACSCanto B (BD Bioscience) and analyzed using FCS Express software, version 7 (*De Novo* Software).

For the characterization of T cells, samples from spleen, lymph nodes, and blood were analyzed by flow cytometry using the following antibodies: anti-CD3 (1:200, APC-Cy7, BioLegend, #100222), anti-CD4 (1:800, BV650, BioLegend, #100469), anti-CD8 (1:1000, BV605, BioLegend, #100744), anti-CD44 (1:800, FITC, BioLegend, #103006), anti-CD62L (1:1000, PE-Cy7, BioLegend, 104418) and anti-CXCR3 (1:100, APC, BioLegend, #126511), anti-CX3CR1 (1:800, BV421, BioLegend, #149023), and TIGIT (1:200, PE, BioLegend, #142103); or anti-CD3 (1:300, FITC, ThermoFisher scientific, #11-0031), anti-CD4 (1:1000, APC, BioLegend, #100516), anti-CD8 (1:400, APC-Cy7, BioLegend, #100713), anti-CCR4 (1:100, BV421, BioLegend, #131218), anti-CCR6 (1:100, BV605, BioLegend, #129819), anti-CXCR5 (1:100, PE-Cy7, BioLegend, #145516) and anti-PD-1 (1:200, PE, BioLegend, #109103).

To identify regulatory T cells, samples from spleen, lymph nodes, and blood were analyzed by flow cytometry using the following antibodies: anti-CD3 (1:300, FITC, ThermoFisher scientific, #11-0031), anti-CD4 (1:800, BV650, BioLegend, #100469), anti-CD8 (1:400, APC-Cy7, BioLegend, #100713), anti-CD25 (1:100, BV480, BD Bioscience, 566120), anti-CD304 (1:100, PE-Cy7, BioLegend, #145211), and anti-CD73 (1:100, BV421, BioLegend, #127217), and intracellularly for anti-FOXP3, (1:40, PE, ThermoFisher scientific, 12-5773-82), and anti-Helios (1:50, APC, BioLegend, #137222).

For thymic T cell identification, samples were analyzed by flow cytometry using the following antibodies: anti-CD4 (1:400, BV650, BioLegend, #100469), anti-CD8 (1:200, APC-Cy7, BioLegend, #100713), anti-CD25 (1:100, APC, eBioscience, 17-0521), anti-CD44 (1:800, FITC, BioLegend, #103006), and anti-CD117 (1:100, PE, BioLegend, #105807).

To measure intracellular cytokines, splenocytes were stimulated with 50 ng/ml phorbol 12-myristate 13-acetate (PMA; Sigma Aldrich, P1585) and 1 μM ionomycin (Sigma Aldrich, I9657), followed by addition of Monensin (BioLegend, 420701) and brefeldin A (BioLegend, 420601) after one hour, for a total of five hours. Samples were analyzed by flow cytometry using the following antibodies: anti-CD3 (1:100, APC-Cy7, BioLegend, #100222), anti-CD4 (1:400, Pacific Blue, BioLegend, #100427), and anti-CD8 (1:1000, BV605, BioLegend, #100744), and intracellularly for anti-IL-4 (1:200, PE, BioLegend, #504104), anti-IL-10 (1:100, APC, ThermoFisher, 17-7101), anti-IFN-γ (1:800, BV785, BioLegend, #505838), and anti-TNF-α (1:800, FITC, BioLegend, 506303).

To identify stem cell populations in the bone marrow, samples were stained with the following antibodies: anti-lineage cocktail (1:5, FITC, ThermoFisher scientific, #22-7770), anti-CD117 (1:100, PE, BioLegend, #105808), anti-Sca-1 (1:100, V500, BD Biosciences, #561228), anti-CD16/32 (1:50, BV711, BioLegend, #101337), anti-CD27 (1:100, BUV395, BD Biosciences, #740247), anti-CD34 (1:50, eFluor450, ThermoFisher scientific, #48-0341), anti-CD48 (1:100, APC-Cy7, BioLegend, #103432), anti-CD127 (1:50, PE-Cy7, ThermoFisher scientific #25-1271), anti-CD135 (1:100, APC, BioLegend, #135310), anti-CD150 (1:100, PerCP-eFluor710, ThermoFisher scientific, #46-1502).

To identify mature cells that returned to the bone marrow, samples were analyzed by flow cytometry using the following antibodies: anti-CD3 (1:200, APC, BioLegend, #100312), anti-CD4 (1:400, BV650, BioLegend, #100469), anti-CD8 (1:400, BV605, BioLegend, #100744), anti-CD19 (1:100, PerCP-Cy5.5, ThermoFisher scientific, #45-0193), anti-CD44 (1:300, FITC, BioLegend, #103006), anti-CD45 (1:100, APC-Cy7, BioLegend, #103115), anti-CD62L (1:800, PE-Cy7, BioLegend, 104418), anti-CD138 (1:200, BV421, BioLegend, #562610), anti-c-kit (1:100, PE, BioLegend, #105808).

For identification of B cell populations, samples were analyzed by flow cytometry using the following antibodies: anti-CD19 (1:200, PE, ThermoFisher scientific, #12-0193), anti-B220 (1:200, APC-eFluor780, eBioscience, 47-0452), anti-CD23 (1:100, BV510, BD Bioscience, #563200), anti-IgM (1:1600, PE-Cy7, ThermoFisher scientific, #25-5790), and either anti-CD95 (1:100, AF647, BD Biosciences, #563647), anti-CD138 (1:100, BV421, BioLegend, #562610) and anti-GL7 (1:100, AF488, ThermoFisher scientific, #53-5902) for splenic and lymph node samples or anti-CD21 (1:100, BV421, BD Bioscience, #562756), anti-CD38 (1:100, FITC, BD Bioscience, #558813) or anti-CD93 (1:100, APC, ThermoFisher scientific, #17-5892) for splenic samples.

For the identification of myeloid cells, samples from spleen and blood were analyzed by flow cytometry using the following antibodies: αCD45 (1:100, APC-Cy7, BioLegend, 103115), anti-CD11b (1:300, PE-Cy7, BD Bioscience, #552850), Ly6G (1:800, FITC, ThermoFisher scientific, #11-5931), Ly6C (1:800, AF647, BioLegend, #128010), Siglec-F (1:100, PE, BD Bioscience, #562068) or anti-CD45 (1:100, APC-Cy7, BioLegend, 103115), anti-CD11b (1:400, FITC, ThermoFisher scientific, #11-0112), anti-CD11c (1:100, eFluor450, ThermoFisher scientific, #48-0114), anti-CD40 (1:100, PE-Cy7, BioLegend, #124621), anti-CD70 (1:100, PE, BioLegend, #104605), anti-CD86 (1:250, APC, ThermoFisher scientific, #17-0862).

### 2.4 Cholesterol levels

Blood was isolated via cardiac puncture and spun down (2100 rpm, 10 minutes, 4°C) to separate plasma from the red blood cells. Total cholesterol was measured by standard enzymatic methods according to the manufacturer’s protocol (CHOD, BIOLABO).

### 2.5 Gene expression

CD4^+^ and CD8^+^ T cells were isolated from the spleen using magnetic activated cell sorting (MACS) according to the manufacturer’s protocol [Miltenyi Biotec, CD4 (L3T3) MicroBeads, mouse, 130-117-043; Miltenyi Biotec, CD8 (Ly-2) MicroBeads, mouse, 130-117-044). Cells were stimulated for two days with plate-bound anti-CD3 (10 ng/ml, Biolegend, 100340)] and cultured in RPMI 1640 medium with HEPES (ThermoFisher Scientific) supplemented with 10% fetal calf serum (FCS) (Gibco), Penicillin-Streptomycin (P/S) (Gibco), 50 µM beta-2-mercaptoethanol (Sigma), 5 ng/ml anti-CD28 (Biolegend, 102116), and 20 ng/ml IL-2 and cell pellet was snap-frozen. Total RNA was isolated using the RNeasy Mini column kit (Qiagen) following the manufacturer’s instructions. RNA concentrations were determined using the Nanodrop 2000 (ThermoFisher). RNA was reverse transcribed using high-capacity cDNA Reverse Transcription kit (Life Technologies, 4368814). Quantitative PCR was performed with SYBR Green PCR kit (Applied Biosystems) on a QuantStudio™ 5 Real-Time PCR system (Applied Biosystems). Gene expression levels were normalized to cyclophilin A and ARBP as reference genes. Primer sequences are available on request.

### 2.6 *In vitro* T cell proliferation and polarization

For *in vitro* experiments, 20-32 weeks old male and/or female mice (age and sex matched per experiment) were used that were fed a normal chow diet. Single cell suspensions of the spleen were retrieved as described above. Samples were first incubated for 5 minutes with anti-CD16/anti-CD32 (1:1000, BioLegend, #101330). Next, cells were stained with anti-CD4 (1:400, BV421, BioLegend, 100443), anti-CD8 (1:800, FITC, eBioscience, 11-0081), anti-CD62L (1:1000, PE-Cy7, BioLegend, 104418), anti-CD44 (1:800, APC, BioLegend, 103012), anti-CD25 (1:400, PE, BioLegend, 102008). Cells were filtered and stained with 7-AAD to exclude dead cells and were sorted using FACS Symphony S6 Cell Sorter (BD Bioscience). For proliferation, sorted CD44^-^CD62L^+^ naive CD4^+^ and CD8^+^ splenic T cells were labelled with 5 µM CFSE (Invitrogen, C34554) according to the manufacturer’s instructions. CFSE-labelled and CFSE-unlabeled control cells were cultured for 3 days (37°C, 5% CO_2_) stimulated with plate-bound CD3 (10 ng/ml) and medium supplemented with 10% FCS, Pen/Strep, 50 µM beta-2-mercaptoethanol, 5 ng/ml anti-CD28, and 20 ng/ml IL-2. Before analysis cells were stained with anti-CD4 (1:1000, APC, BioLegend, #100516), anti-CD8 (1:400, APC-Cy7, BioLegend, #100713), and DAPI was used to exclude dead cells. Fluorescence was measured on Symphony A1 Cell Analyzer (BD Bioscience).

For polarization, RPMI 1640 medium containing 10% FCS, Pen/Strep, 50 µM beta-2-mercaptoethanol, 5 ng/ml anti-CD28, and 20 ng/ml IL-2 was supplemented with 10ug/ml IL-12/IL-23 neutralizing antibody (BioLegend, 505308) for Th0 polarization, and supplemented with 20 ng/ml IL-12 (R&D systems, 419-ML) for Th1 polarization. Cells were cultured for 5 days on a CD3 coated (10 ng/ml) plate at 37°C, 5% CO_2_. Before analysis the cells were stained as described before, with anti-CD4 (1:1000, APC, BioLegend, #100516), anti-IFN-γ (1:800, BV786, BioLegend, #505838). Fluorescence was measured on Symphony A1 Cell Analyzer (BD Bioscience).

### 2.7 Statistics

Data are presented as mean ± SD. Statistical analysis were performed using GraphPad Prism 9.0 software. Outliers were removed by ROUT test (Q=1%). Normality was assessed by Shapiro-Willk normality test. Normally distributed data are analyzed by unpaired t-test, while non-normally distributed data are analyzed by Mann-Whitney U test, unless otherwise specified. * p < 0.05, ** p < 0.01, *** p < 0.001, **** p < 0.0001.

## 3 Results

### 3.1 Cbl-b^cKO^ mice

The knock-down efficiency of T cell CBL-B was 90% in activated CD4^+^ and 80% in activated CD8^+^ T cells (**Supplementary Figures 1A, B**). The weight of the mice was not significantly different between the groups (**Supplementary Figure 1C**). Autopsy showed no apparent macroscopic differences in the visceral organs as well as no apparent microscopic differences in the spleen, lymph nodes, and thymus. There were no signs of autoimmunity, including no signs of adenopathy, and the spleen weight was similar between groups (**Supplementary Figure 1D**). Female *Apoe^-/-^Cd4^cre^Cblb^f/lfl^* (Cbl-b^cKO^) and *Apoe^-/-^ Cd4^WT^Cblb^f/lfl^* (Cbl-b^fl/fl^) (6-8 weeks) mice were fed a high cholesterol diet (HCD) for ten weeks. Cholesterol levels were similar in Cbl-b^cKO^ and Cbl-b^fl/fl^ mice after ten weeks HCD (**Supplementary Figure 1E**).

### 3.2 Cbl-b^cKO^ mice have smaller atherosclerotic plaques that contain less collagen

**Figure 1:**
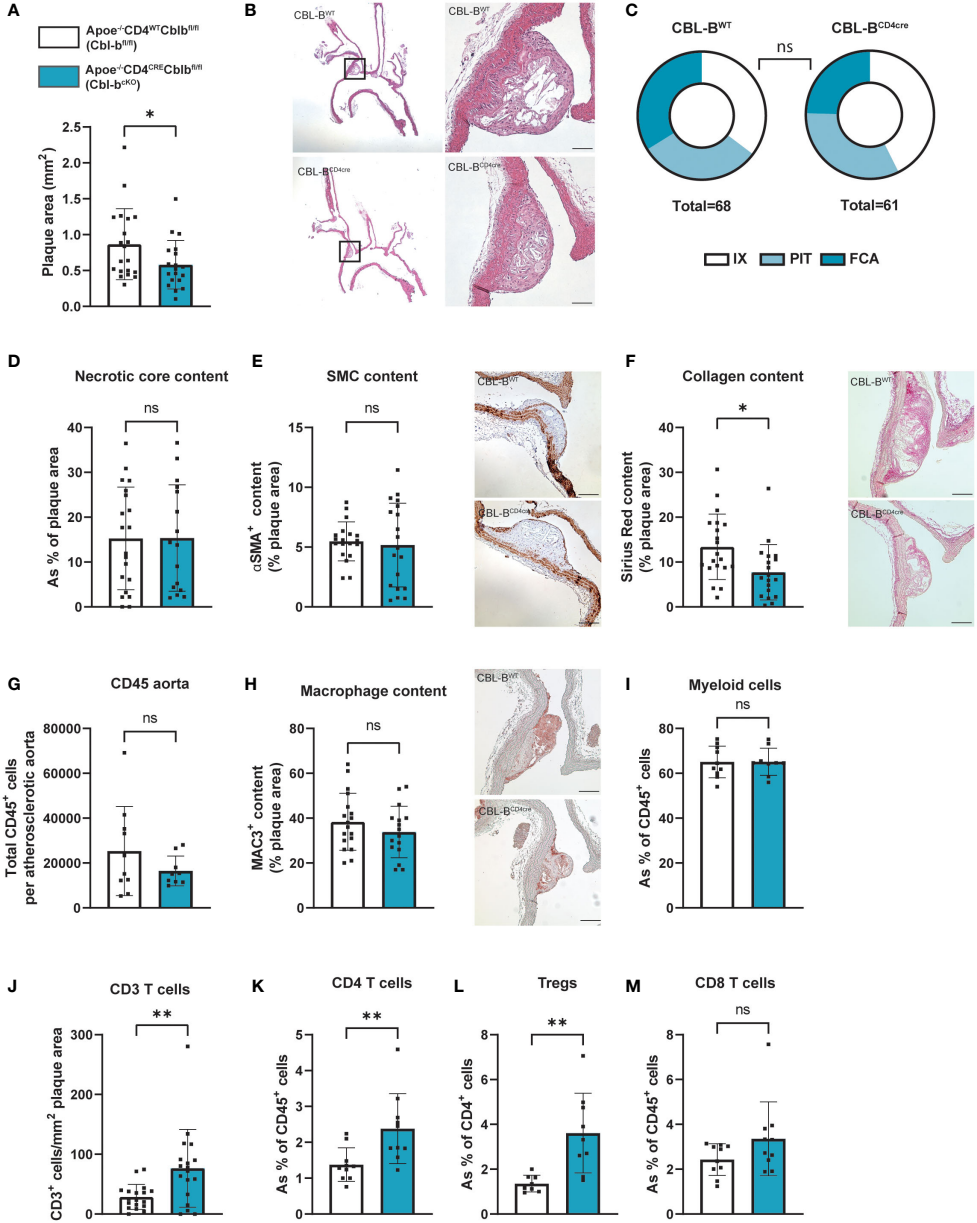
Cbl-b^cKO^ mice have smaller atherosclerotic plaque size, with increased T cell infiltration. **(A)** Atherosclerotic plaque area in the aortic arch including its main branch points of Cbl-b^fl/fl^ (n=20) and Cbl-b^cKO^ mice (n=19) after 10 weeks of HCD. **(B)** Representative longitudinal section of aortic arches (haematoxylin and eosin staining, scale bar represents 100 µm). **(C)** Virmani classification of the plaques in the aortic arch, categorised in either intimal xanthoma (IX), pathological intimal thickening (PIT), or fibrous cap atheroma (FCA). **(D)** Quantification of necrotic core area in plaques of the aortic arch (n=20/16). **(E)** Quantification and representative images (scale bar represents 100 µm) of plaque smooth muscle cell (SMC) content (αSMA^+^, n=19/19) in the aortic arch. **(F)** Histochemical quantification and representative images (scale bar represents 100 µm) of collagen content (Sirius Red, n=20/19). **(G)** Total CD45^+^ cells per atherosclerotic aorta as measured by flow cytometry (n=10/10). **(H)** Immunohistochemical quantification and representative images (scale bar represents 100 µm) of plaque of macrophage (MAC3^+^, n=18/16) content. **(I)** The percentage of CD11b^+^ myeloid cells from the total CD45^+^ population in the atherosclerotic aorta (n=10/10). **(J)** Immunohistochemical quantification of the number of CD3^+^ T cells in the aortic arch (n=18/18). The percentage of CD4^+^ T cells **(K)**, CD4^+^CD25^+^FoxP3^+^ Tregs **(L)**, and CD8^+^ **(M)** T cells in the atherosclerotic aorta (n=10/10) as measured by flow cytometry. Data is shown as mean ± SD, outliers were removed by ROUT test (Q = 1%) and normality was tested Shapiro-Wilk normality test. Normally distributed data was analysed by an unpaired 2-tailed student t-test and non-normally distributed data was analysed by Mann-Whitney U test. Statistical significance is displayed as *p < 0.05, **p < 0.01, not significant (ns).

Plaque size and phenotype were determined at two sites, in the aortic arch including its main branch points and in the aortic root. In the aortic arch, plaque area of Cbl-b^cKO^ mice was reduced by 30% compared to Cbl-b^fl/fl^ mice (**Figures 1A, B**). No significant differences in plaque stage could be detected between Cbl-b^cKO^ and Cbl-b^fl/fl^ mice (Chi-square test, p=0.49) (31) (**Figure 1C**). Overall, plaques in the aortic arch were early, macrophage rich lesions: Cbl-b^cKO^ had 66% initial and intermediate plaques [intimal xanthomas (IX) + pathological intimal thickening (PIT)] and 34% advanced plaques [Fibrous cap atheroma (FCA)]. In Cbl-b^fl/fl^ mice, 75% of the plaques had an initial and intermediate phenotype and 25% of the plaques were classified as advanced plaque. Necrotic core content in the plaques of the brachiocephalic artery (**Figure 1D**), and αSMA^+^ vascular smooth muscle cell content (**Figure 1E**) were unaffected by T cell specific CBL-B deficiency. However, collagen content was decreased in plaques of Cbl-b^cKO^ mice (**Figure 1F**). The aortic root showed a plaque phenotype similar, but less pronounced, than in the aortic arch, with a 12% reduction in plaque size (**Supplementary Figure 1F**). Necrotic core content, αSMA^+^ vascular smooth muscle cell content, and collagen content were not affected by T cell CBL-B deficiency in the aortic root (**Supplementary Figures 1G–K**).

### 3.3 Cbl-b^cKO^ mice have increased T cell numbers in the atherosclerotic plaque, blood, and spleen

**Figure 2:**
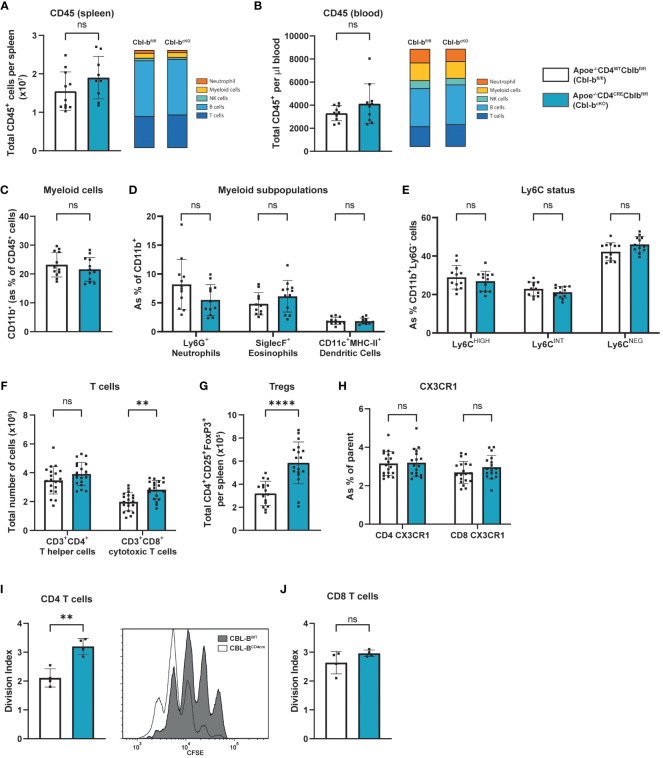
Cbl-b^cKO^ mice have more T cells, while myeloid cells are not affected. Absolute number of CD45^+^ leukocytes (n=10/10) and relative amount of major immune cell populations in the spleen **(A)** and blood **(B)**. **(C)** Percentage of CD11b^+^ myeloid cells in the circulation (n=12/12). **(D)** Frequency of Ly6G^+^ neutrophils (n=12/12), siglecF^+^ eosinophils (n=11/12), and CD11c^+^MHC-II^+^ dendritic cells (n=11/11). **(E)** Ly6C expression level of CD11b^+^Ly6G cells in the circulation (n=12/12). Absolute number of **(F)** CD4^+^ and CD8^+^ T cells (n=20/20), and **(G)** CD25^+^FoxP3^+^ regulatory T cells (n=16/18) in the spleen. **(H)** Frequency of CX3CR1 expressing of CD4^+^ T cells (n=14/15) or CD8^+^ T cells (n=17/15) in the circulation. Division index of **(I)** CD4^+^ and **(J)** CD8^+^ T cells after 3-day CD3/ CD28/IL-2 induced proliferation with representative image of CFSE proliferation flow cytometric staining of CD4^+^ T cells (n=4/4). Data is shown as mean ± SD, outliers were removed by ROUT test (Q = 1%) and normality was tested Shapiro-Wilk normality test. Normally distributed data was analysed by an unpaired 2-tailed student t-test and non-normally distributed data was analysed by Mann-Whitney U test. Statistical significance is displayed as **p < 0.01, ****p < 0.0001, not significant (ns).

The absolute number of CD45^+^ cells as measured by flow cytometry in the atherosclerotic aorta was similar between the Cbl-b^cKO^ and Cbl-b^fl/fl^ mice (**Figure 1G**). Plaque MAC3^+^ content determined by immunohistochemistry was similar in the aortic arch (**Figure 2H**) and in the aortic root (**Supplementary Figure 1J**), as well as the total number of CD11b^+^ myeloid cells in the atherosclerotic aorta, as determined by flow cytometry (**Figure 1I**). Plaques of Cbl-b^cKO^ mice had increased CD3^+^ T cell numbers in the aortic arch (2.2-fold) (**Figure 1J**) and in the aortic root (1.4-fold), shown by immunohistochemistry staining for CD3 (**Supplementary Figure 1K**). Using flow cytometry, we demonstrated that relative numbers of CD4^+^ T cells and CD4^+^CD25^+^FoxP3^+^ Tregs were increased, while CD8^+^ T cell numbers were similar in the plaques of Cbl-b^cKO^ mice compared to Cbl-b^fl/fl^ mice. (**Figures 1K–M**).

Splenic and circulating CD45^+^ leukocyte numbers were unaltered in Cbl-b^cKO^ mice compared to Cbl-b^fl/fl^ mice, similar as seen in the atherosclerotic plaque (**Figures 2A, B**). Cbl-b^cKO^ mice did not have altered numbers of myeloid cells in circulation (**Figure 2C**), including neutrophils, eosinophils, and dendritic cells (**Figure 2D**), or the spleen (**Supplementary Figure 2A**). Moreover, CBL-B specific T cell deficiency did not affect the percentages of Ly6C^high^ classical or Ly6C^low^ non-classical monocytes, (**Figure 2E**, **Supplementary Figure 2B**). Cbl-b^cKO^ mice did not show alterations in total B cell numbers in the spleen and lymph node, including memory B cells, germinal center (GC) B cells, and plasma cells (**Supplementary Figures 2C–G**). In the spleen, lymph nodes and circulation, CD8^+^ T cell numbers were increased, while CD4^+^ T cell numbers were not affected (**Figure 2F**; **Supplementary Figures 2H–J**). Moreover, we found an 1.8-fold increase in the absolute number of Tregs in the spleens of Cbl-b^cKO^ mice compared to the Cbl-b^fl/fl^ mice (**Figure 2G**; **Supplementary Figure 2K**), while Treg numbers in the lymph nodes and blood were not affected (**Supplementary Figures 2L, M**). There are no changes in the expression of Helios, Neuropilin 1 (Nrp1) and CD73, and FoxP3 (32, 33), between Tregs of Cbl-b^cKO^ and Cbl-b^fl/fl^ mice (**Supplementary Figures 2N–P**). Next, we measured the fractalkine receptor CX3CR1, which is known to mediate migration, adhesion, and retention of leukocytes towards the inflamed vascular wall (34). We observed no differential expression of CX3CR1 in Cbl-b^cKO^ and Cbl-b^fl/fl^ in either CD4^+^ or in CD8^+^ T cells, indicating that vascular homing of T cells is not affected by CBL-B (**Figure 2H**). CBL-B deficient T cells are known to proliferate faster (25, 26), and, consistent with those reports, we observe that Cbl-b^cKO^ CD4^+^ proliferate faster compared to Cbl-b^fl/fl^ CD4^+^ T cells, while proliferation of CD8^+^ T cells in Cbl-b^cKO^ and Cbl-b^fl/fl^ was comparable (**Figures 2I, J**).

### 3.4 CBL-B T cell deficiency increases T cell activation

**Figure 3:**
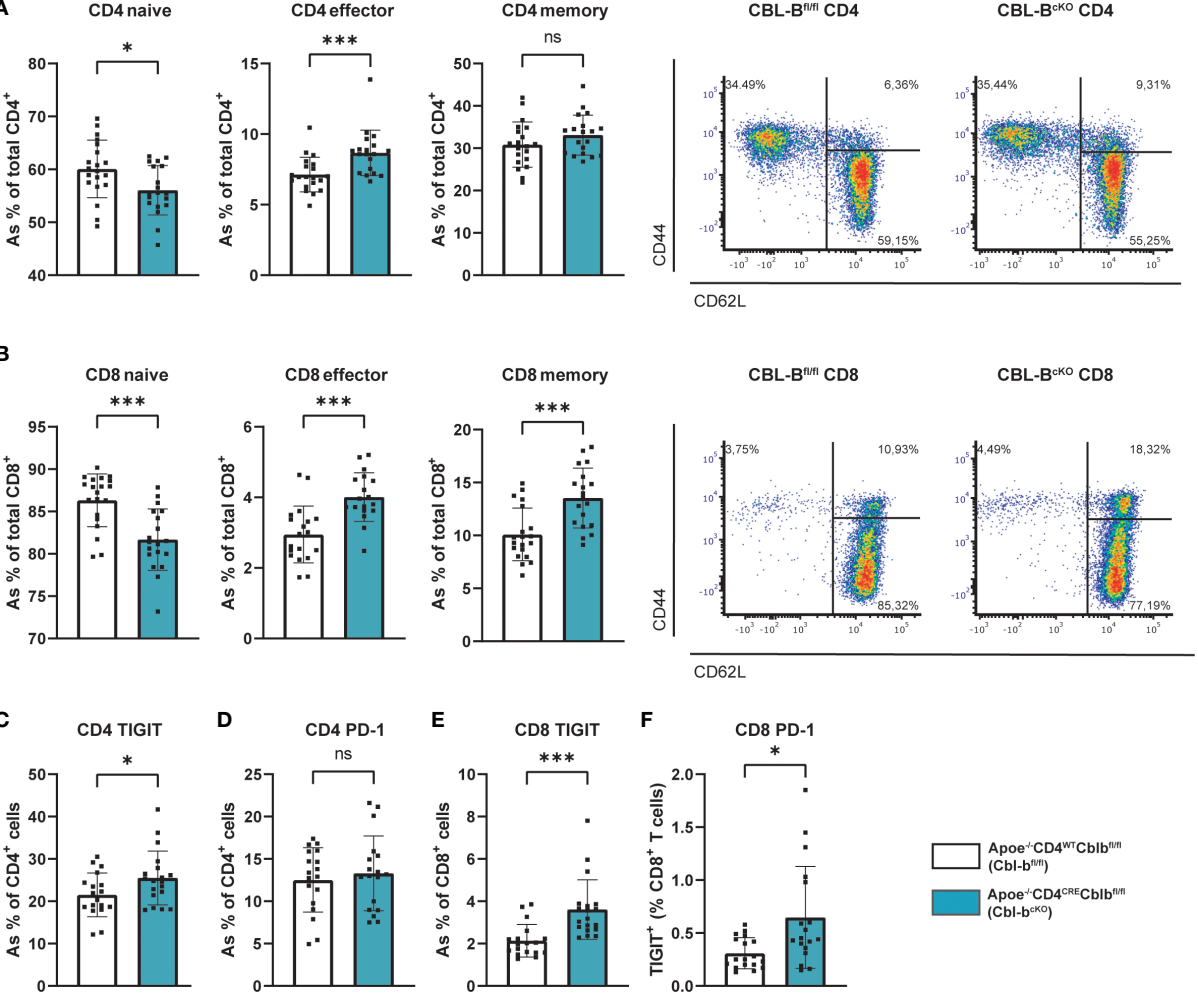
Cbl-b^cKO^ mice have increased T cell activation and exhaustion. Percentage of naive, effector, and central memory cells in the **(A)** splenic CD4^+^ T cell population (n=20/19) and the **(B)** splenic CD8^+^ T cell population (n=20/19), including representative dot plot of the flow cytometric analysis of T cell activation. Quantification of **(C)** TIGIT expression (n=18/19) and **(D)** PD-1 expression (n=18/19) of splenic CD4^+^ T cells. Quantification of **(E)** TIGIT expression of CD8^+^ T cells in spleen (n=19/18) and **(F)** PD-1 expression (n=18/18). Data is shown as mean ± SD, outliers were removed by ROUT test (Q = 1%) and normality was tested Shapiro-Wilk normality test. Normally distributed data was analysed by an unpaired 2-tailed student t-test and non-normally distributed data was analysed by Mann-Whitney U test. Statistical significance is displayed as *p < 0.05, ***p < 0.001, not significant (ns).

In the absence of T cell specific CBL-B, the T cell subset ratio in CD4^+^ splenic T cells shifted from a naive (CD44^-^CD62L^+^) to an effector memory (CD44^+^CD62L^-^) phenotype, indicating their enhanced activation (**Figure 3A**). In the CD8^+^ T cells this phenotype was similar, with a shift from naive to effector and central (CD44^+^CD62L^+^) memory phenotype (**Figure 3B**). In the circulation and the lymph nodes, the activated T cell phenotype was less pronounced, but shows a similar trend. (**Supplementary Figures 3A–D**). Upon T cell activation, immune checkpoints are expressed, which regulate immune activation and inhibition. High expression of co-inhibitory immune checkpoints, such as programmed cell death protein (PD-1) and T cell immunoreceptor with Ig and ITIM domains (TIGIT), can be a sign of T cell exhaustion (35). In the splenic CD4^+^ T cells of Cbl-b^cKO^ mice, we observed an increase in the inhibitory immune checkpoints PD-1, but not in TIGIT (**Figures 3C, D**), while CBL-B deficient splenic CD8^+^ T cells showed increased expression of both TIGIT and PD-1 (**Figures 3E, F**). Altogether, Cbl-b^cKO^ T cells have an effect or phenotype and show markers associated with T cell exhaustion. These effects were more pronounced in CD8^+^ T cells.

The CD4-cre driver was found to induce gene deletion in a small proportion of hematopoietic stem cells (30), and deletion of CBL-B in these hematopoietic cells may affect T cell development and maturation at an early stage Therefore, we evaluated leukocyte development in the bone marrow and T cell maturation in the thymus in our Cbl-b^cKO^ mice. We observed no differences in short-term stem cells (ST-SC), long-term stem cells (LT-SC), and multipotent progenitors (MPP) between Cbl-b^cKO^ and Cbl-b^fl/fl^ mice (**Supplementary Figures 4A, B**). In the common lymphoid progenitor (CLP) population, we observed an increase in the late CLP in T cell specific CBL-B deficient mice (**Supplementary Figure 4C**). Moreover, we observed an increase in mature CD8^+^ T cells that have returned to the bone marrow (**Supplementary Figure 4D**). However, here, we did not observe any differences in both CD4^+^ and CD8^+^ T cell activation in the bone marrow (**Supplementary Figures 4E, F**). In the thymus, no differences were observed in the double negative stages (DN1-4), the double positive stage (DP), and the single positive stage (SP), indicating that, overall, Cbl-b^cKO^ T cells develop normally (**Supplementary Figures 4G, H**). These data shows that the increased T cell numbers originate mainly in the secondary lymphoid organs, and the majority of the effects of CBL-B on T cells become apparent after T cell activation.

### 3.5 Cbl-b^cKO^ mice have an enhanced pro-inflammatory T cell phenotype

**Figure 4:**
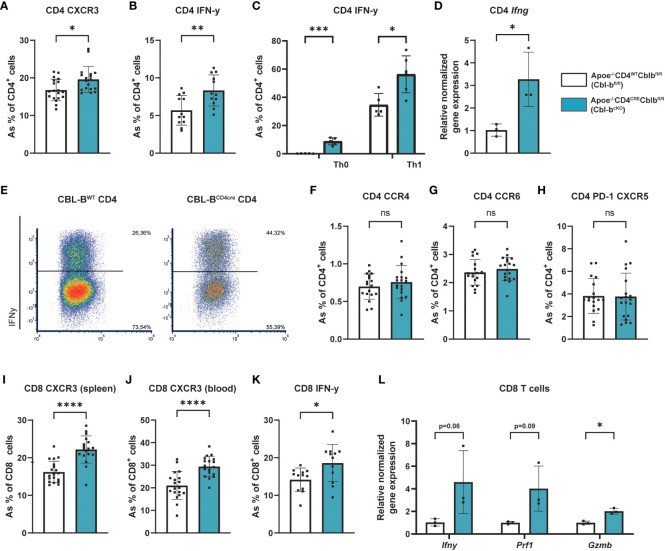
Cbl-b^cKO^ T cells have a more pro-inflammatory phenotype. **(A)** Frequency of CXCR3 expressing splenic Th1 cells (n=19/18). **(B)** Quantification of IFN-γ producing splenic CD4^+^ T cells (n=12/12) after five hours of PMA/Ionomycin stimulation, with Monensin and Brefeldin added after the first hour. **(C)** Quantification of IFN-γ producing splenic CD4^+^ T cells after 5-day Th0 and Th1 polarization (n=5/5), including representative dot plot of the flow cytometric analysis of IFN-γ production by Th1 cells after 5-day polarization **(E)**. **(D)** Relative normalised gene expression of Ifng in CD4^+^ T cells after 2-day stimulation with CD3/CD28/IL-2 (n=3/3). Percentage of **(F)** Th2 (CCR4^+^), **(G)** Th17 (CCR6^+^), and **(H)** T follicular helper (PD-1^+^CXCR5^+^) cells (n=18/19). Frequency of CXCR3 expressing splenic CD8^+^ T cells (n=19/18) in **(I)** spleen and **(J)** blood. **(K)** Quantification of IFN-γ producing splenic CD8^+^ T cells (n=12/12) after five hours of PMA/Ionomycin stimulation, with Monensin and Brefeldin added after the first hour. **(L)** Relative normalised gene expression of IFN-γ, perforin 1 (Prf1), and Granzyme B (Gzmb) in CD8^+^ T cells after 2-day stimulation with CD3/CD28/IL-2 (n=3/3). Data is shown as mean ± SD, outliers were removed by ROUT test (Q = 1%) and normality was tested Shapiro-Wilk normality test. Normally distributed data was analysed by an unpaired 2-tailed student t-test and non-normally distributed data was analysed by Mann-Whitney U test. Statistical significance is displayed as *p < 0.05, **p < 0.01, ***p < 0.001, ****p < 0.0001, not significant (ns).

While the absolute number of CD4^+^ T cells in the spleen was unaffected, CD4^+^ splenic T cells from Cbl-b^cKO^ mice shifted to a pro-inflammatory Th1 phenotype, as indicated by increased expression of CXCR3 and IFN-γ (**Figures 4A, B**). Interestingly, Cbl-b^cKO^ CD4^+^ T cells already produced more IFN-γ in IL-2 stimulated Th0 conditions (without polarizing cytokines), underlining the pro-inflammatory status of Cbl-b^cKO^ CD4^+^ T cells (**Figure 4C**). In line with the Th1 phenotype, Cbl-b^cKO^ CD4^+^ T cells produced more IFN-γ after Th1 polarization with IL-12 compared to Cbl-b^fl/fl^ CD4^+^ T cells (**Figures 4C, E**). After a two-day activation with anti-CD3, anti- CD28, and IL-2, CD4^+^ T cells also had increased *Ifng* gene expression (**Figure 4D**). We observed no differences in other splenic T cell subsets, including Th2 (CCR4), Th17 (CCR6), and T follicular helper (Tfh) (PD1, CXCR5) (**Figures 4F–H**). Similar to the CD4^+^ T cells, splenic and circulating CD8^+^ T cells had increased CXCR3 and IFN-γ expression (**Figures 4I–K**), hallmarks of effector CD8^+^ T cells. After activation, Cbl-b^cKO^ CD8^+^ T cells had increased expression of *Ifng*, *Prf1*, and *Gzmb*, indicating that CD8^+^ effector molecules were increased (**Figure 4L**). These data show that CBL-B deficient mice specifically have increased pro-inflammatory CD4^+^ and CD8^+^ T cells.

## 4 Discussion

In the current study, we report that Cbl-b^cKO^ mice have smaller atherosclerotic lesions, with an increased number of T cells and decreased collagen content in the plaque compared to Cbl-b^fl/fl^ mice. In the spleen, T cell CBL-B deficiency induced an activated and pro-atherogenic T cell profile, by switching from a naive to a memory phenotype and by increasing the expression of the effector molecules CXCR3 and IFN-γ, eliciting a Th1-like/effector CD8^+^ T cell response. Moreover, CBL-B deficient T cells show markers of T cell exhaustion, i.e. increased expression of the inhibitory immune checkpoints TIGIT and PD-1.

The reduction in lesion size in Cbl-b^cKO^ mice was associated with a decrease in collagen deposition in atherosclerotic plaques. T cell driven inflammation can affect collagen maturation in the atherosclerotic plaque by altering the collagen crosslinking in the extracellular matrix (36). Similarly, another study showed that in *Apoe* ^-/-^ mice, Granzyme B reduced the collagen content in the plaque, due to Granzyme B degrading proteoglycans in the extracellular matrix (37, 38). Granzyme B is abundant in advanced plaques, and it is linked to a more vulnerable plaque phenotype (39, 40). In our study, we show that after anti-CD3/CD28 activation, CBL-B deficient CD8^+^ splenic T cells have increased Granzyme B expression, which may lead to increased collagen breakdown and could explain the reduction in collagen observed in plaques of Cbl-b^cKO^ mice.

In our previous study, we reported that full deletion of CBL-B leads to larger and more advanced atherosclerotic plaques in *Apoe^-/-^* mice, by inducing CD8^+^ T cell-mediated macrophage apoptosis (29). To our surprise, CBL-B deficiency in T cells resulted in significantly smaller plaques. Although CBL-B is extensively studied in T cells, it is also highly expressed in macrophages (41). CBL-B functions as a negative regulator of macrophage recruitment and activation (42), and CBL-B deletion results in more pro-inflammatory macrophages (29). Activated macrophages produce chemokines, that recruit cells to the vessel wall, thereby contributing to the development and progression of atherosclerosis. For example, CCR2 is a major chemokine which is highly expressed on macrophages, and mice lacking CCR2 show a reduction in atherosclerotic lesion size (43). CBL-B suppresses macrophage migration by interfering in CCR2-Vav1 signaling, through inhibition of the phosphorylation of Vav1 (44). In the current study, we observed that T cell specific CBL-B depletion had no effect on both macrophage activation in the atherosclerotic lesion and peripheral macrophages/myeloid cells, indicating that there are no (in)direct effects of Cbl-b^cKO^ on other immune cells than T cells. The lack of the initial trigger given by macrophages in the progression of the atherosclerosis could be an explanation for the difference in plaque size and phenotype between the full body CBL-B knock-out and the T cell specific CBL-B deficiency. This is further supported by the fact that we observe no differences in the expression of the chemokine receptor CX3CR1, which is used by T cells to infiltrate the plaque (45). This suggests that the increased number of T cells in the plaque is not an effect of increased T cell infiltration, but possibly an increase in T cell proliferation in situ, as we see that Cbl-b^cKO^ CD4^+^ T cells proliferate faster compared to Cbl-b^fl/fl^ CD4^+^ T cells in culture. This supports earlier research, which shows CBL-B deficient T cells proliferate faster due to increased IL-2 production after antigen stimulation (25, 26). Altogether, the effect of CBL-B deficient T cells on the progression of the plaque is limited to a decrease in collagen content, and the role of CBL-B in macrophages might be more significant in atherosclerosis progression than initially hypothesized. Future experiments to study the macrophage specific role of CBL-B in atherosclerosis will include the generation of a macrophage specific, CBL-B deficient mouse model mouse (e.g. *Apoe* ^-/-^ *LysM* ^cre^ *Cbl-b* ^fl/fl^) or macrophage depletion studies in which we reconstitute *ApoE^-/-^* mouse with CBL-B deficient versus wild type macrophages.

CBL-B deficient T cells are hyper-responsive to antigen stimulation, characterized by an increased cytokine production, and resistance to Treg suppression (28, 29, 46). Moreover, CBL-B deficient T cells are reported to be less vulnerable to anergic signals (27, 28), leading to prolonged T cell activation, which may eventually result in T cell exhaustion. T cell exhaustion is characterized by increased expression of inhibitory receptors, a reduction in their proliferative capacities, and overall impaired effector functions (47). In CBL-B deficient CD8^+^ T cells, we observed an increased expression of the inhibitory checkpoints TIGIT and PD-1, as well as reduced proliferative capacities after prolonged T cell activation *in vitro*. Together, this indicates an exhausted-like CD8^+^ T cell phenotype in Cbl-b^cKO^ mice, which could potentially explain the limited effect of T cells on plaque composition that we observed (48, 49). Moreover, besides their suppressive capacities, Tregs can promote T cell exhaustion by secretion of IL-10 and IL-35, which may induce the expression of inhibitory receptors such as PD-1, TIM3, and LAG3 (50). The vast increase of splenic Tregs in Cbl-b^cKO^ mice could advance T cell exhaustion, thereby contributing to overall dysfunction of CBL-B deficient T cells. Altogether, CBL-B deficient T cells show hints of an exhausted phenotype, which could result in their limited effect in the plaque. Exhausted T cells lose some of their effector functions, and thereby potentially also their pro-atherogenic role. For example, stimulation of the main immune checkpoint for T cell exhaustion, PD-1, decreases the formation of atherosclerotic lesions in mice by reducing T cell activation and proliferation (51). Moreover, Bazioti et al. show that increased T cell senescence, resulting in defects in proliferation and effector functions, can lead to a reduction of atherosclerosis in *Ldlr^-/-^* mice (52). These earlier results are in line with our study in which we show that CBL-B deficient T cells are hyper-responsive to antigen stimulation, leading to CD8^+^ T cell dysfunction, and resulting in a lower atherosclerotic burden. While increased T cell numbers in the plaque are often associated with increased macrophage death and larger necrotic core (20, 53), we see no effect on macrophage death and necrotic core size. This could partially be the result of CBL-B deficient dysfunctional T cells producing less effector molecules, thereby not affecting MAC3^+^ macrophage content and necrotic core size.

Altogether, we show that a T cell specific CBL-B deficiency increases T cell numbers in the plaque and lymphoid organs and increases their activation and polarization towards a more pro-inflammatory phenotype in *Apoe* ^-/-^ mice subjected to a high cholesterol diet. However, continuous antigen stimulation in the atherosclerotic plaque may induce T cell dysfunction, which may limit T cell-driven inflammation in the plaque.

## Data availability statement

The raw data supporting the conclusions of this article will be made available by the authors, without undue reservation.

## Ethics statement

The animal study was approved by Committee of Animal Welfare of the University of Amsterdam. The study was conducted in accordance with the local legislation and institutional requirements.

## Author contributions

WV: Conceptualization, Data curation, Formal analysis, Investigation, Methodology, Writing – original draft, Writing – review & editing. BO: Data curation, Investigation, Writing – review & editing, Writing – original draft. MT: Investigation, Writing – review & editing. LB: Investigation, Writing – review & editing. CR: Investigation, Writing – review & editing. CT: Investigation, Writing – review & editing. BM: Resources, Writing – review & editing. HB: Resources, Writing – review & editing. KN: Resources, Writing – review & editing. CW: Funding acquisition, Writing – review & editing. DA: Funding acquisition, Resources, Writing – review & editing. MW: Supervision, Writing – review & editing. LAB: Data curation, Investigation, Supervision, Writing – review & editing, Writing – original draft. EL: Conceptualization, Funding acquisition, Supervision, Writing – original draft, Writing – review & editing. TS: Conceptualization, Funding acquisition, Supervision, Writing – original draft, Writing – review & editing.

## Supplementary material

Supplementary Figure 1 Relative normalized gene expression of *Cblb* in **(A)** CD4^+^ T cells and **(B)** CD8^+^ T cells (n=3/3). **(C)** Weight of the mice after 10 weeks of high cholesterol diet (n=11/10). **(D)** Spleen weight as percentage of body weight after 10 weeks of high cholesterol diet (n=10/8). **(E)** Cholesterol levels of CBL-B^fl/fl^ (n=20) and Cbl-b^cKO^ (n=19) mice after 10 weeks of high cholesterol diet. **(F)** Atherosclerotic plaque volume in the aortic root of CBL-B^fl/fl^ (n=20) and Cbl-b^cKO^ mice (n=19)**. (G)** Quantification of necrotic core area in plaques of the aortic root (n=20/19). **(H)** Immunohistochemical quantification of plaque smooth muscle cell content (αSMA^+^, n=20/18) in the aortic root. **(I)** Histochemical quantification of collagen content (Sirius Red, n=19/17). **(J)** Immunohistochemical quantification of plaque macrophage (MAC3^+^, n=20/17) content in the aortic root. **(K)** Quantification of the number of CD3^+^ (n=20/17) T cells in the aortic root. Data is shown as mean ± SD, outliers were removed by ROUT test (Q = 1%) and normality was tested Shapiro-Wilk normality test. Normally distributed data was analysed by an unpaired 2-tailed student t-test and non-normally distributed data was analysed by Mann-Whitney U test. Statistical significance is displayed as * p < 0.05, ** p < 0.01, *** p < 0.001, not significant (ns).

Supplementary Figure 2
**(A)** Percentage of CD11b+ myeloid cells (n=12/12), Ly6G+ neutrophils (n=12/12), siglecF+ eosinophils (n=11/12), and CD11c+MHC-II+ dendritic cells (n=12/11) in the spleen. **(B)** Ly6C expression level of CD11b+Ly6G- cells in the circulation (n=12/12). **(C)** Percentage of CD19+B220+ B cells in the spleen. **(D)** Frequency of CD21+CD23-IgM+ memory B cells (n=12/12), and GL7+ germinal center (GC) B cells (n=12/11). **(E)** Frequency of CD138+IgM+ plasma cells (n=12/11). Frequency of **(F)** B2 cells (n=12/12) and **(G)** plasma cells (n=12/12) in the lymph node. Percentage of CD4+ and CD8+ T cells in **(H)** spleen (n=20/20), **(I)** lymph node (LN), and blood **(J)** (n=19/20). Percentage of Tregs cells in **(K)** spleen (n=16/18), **(L)** lymph node (LN) (n=19/19), and **(M)** blood (n=16/18). Expression of **(N)** Helios and **(O)** double expression of Nrp1 and CD73, and **(P)** FoxP3 mean fluorescence intensity (MFI) in Tregs cells in the spleen (n=16/18). Data is shown as mean ± SD, outliers were removed by ROUT test (Q = 1%) and normality was tested Shapiro-Wilk normality test. Normally distributed data was analysed by an unpaired 2-tailed student t-test and non-normally distributed data was analysed by Mann-Whitney U test. Statistical significance is displayed as * p < 0.05, **** p < 0.0001., not significant (ns)

Supplementary Figure 3 Percentage of naive, effector, and central memory in the **(A)** blood and **(C)** lymph node CD4+ T cell population (n=20/18) and the **(B)** blood (n=19/19) and **(D)** lymph node (n=20/18) CD8+ T cell population. Data is shown as mean ± SD, outliers were removed by ROUT test (Q = 1%) and normality was tested Shapiro-Wilk normality test. Normally distributed data was analysed by an unpaired 2-tailed student t-test and non-normally distributed data was analysed by Mann-Whitney U test. Statistical significance is displayed as * p < 0.05, ** p < 0.01, not significant (ns).

Supplementary Figure 4
**(A–C)** Flow cytometric analysis of stem cell population in the bone marrow (n=12/12). **(D)** Frequency of CD4+ and CD8+ T cells that have returned in the bone marrow (n=12/12). Percentage of naive, effector, and central memory in the **(E)** CD4+ T cell population and the **(F)** CD8+ T cell population in the bone marrow (n=12/12). **(G, H)** Developmental stages of T cells in the thymus (n=12/12). Statistical significance is displayed as *** p < 0.001, **** p < 0.0001, not significant (ns).

Supplementary Table 1 Primers used for genotyping.
