# Supplementary material for: T cell specific deletion of Casitas B lineage lymphoma-b reduces atherosclerosis, but increases plaque T cell infiltration and systemic T cell activation
Source: Front Immunol. 2024 Mar 4;15:1297893. doi: 10.3389/fimmu.2024.1297893 (PMC10949527; doi:10.3389/fimmu.2024.1297893)
Supplement: Supplementary file 1 [file Table_1.docx]

# Supplementary Table

Table 1: Primers used for genotyping

| Cblb_floxed_FW | TTCTTTTACACGGCTATCAAGGAC |
| --- | --- |
| Cblb-floxed_RV | CAAATAAAGAGCAGCAGTCCCATA |
| CD4cre_FW | CGATGCAACGAGTGATGAGG |
| CD4cre_RV | GCATTGCTGTCACTTGGTCCT |
