# Supplementary figures and images for: T cell specific deletion of Casitas B lineage lymphoma-b reduces atherosclerosis, but increases plaque T cell infiltration and systemic T cell activation
Source: Front Immunol. 2024 Mar 4;15:1297893. doi: 10.3389/fimmu.2024.1297893 (PMC10949527; doi:10.3389/fimmu.2024.1297893)

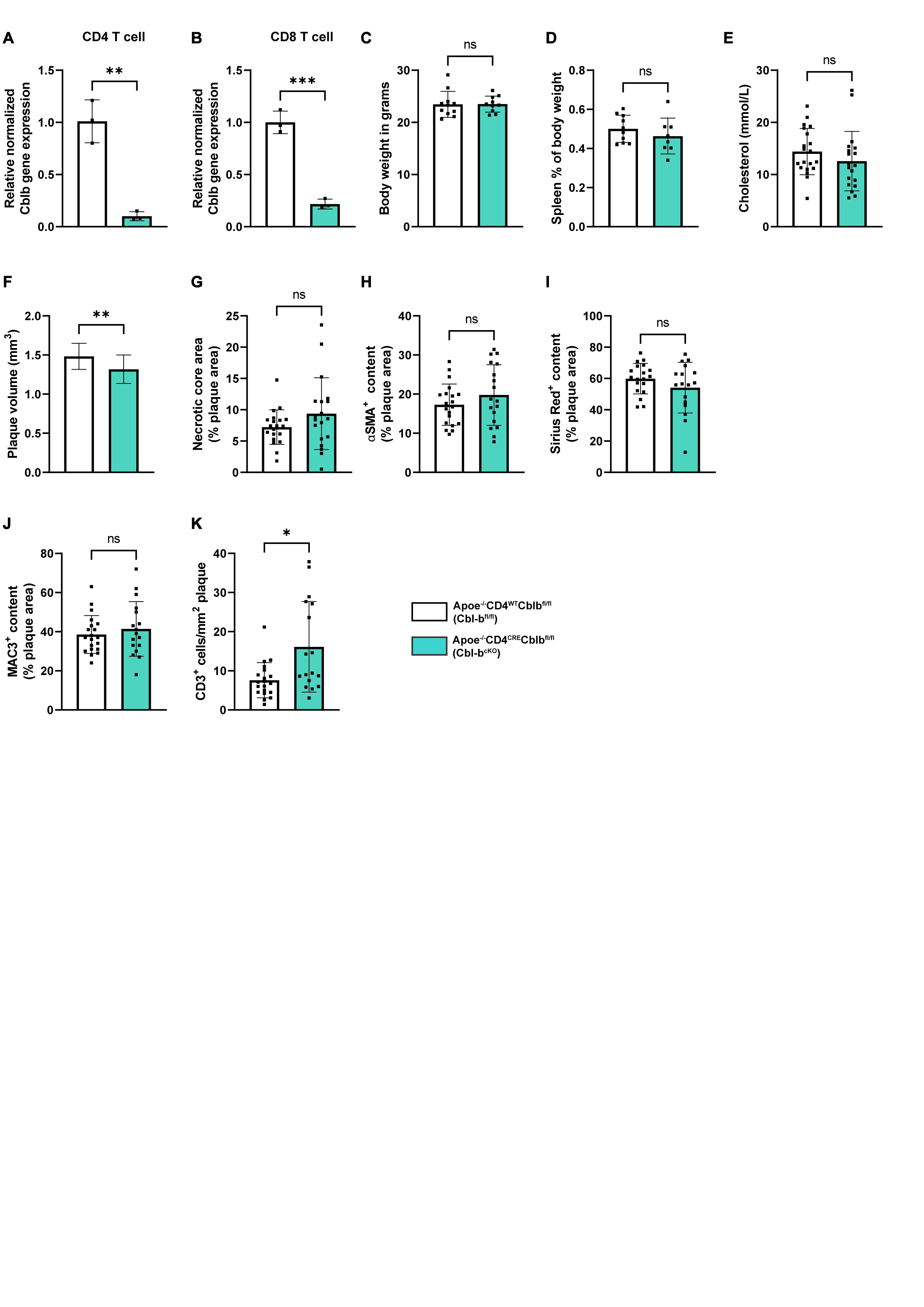

Supplement: Supplementary Figure 1 — Relative normalized gene expression of Cblb in (A) CD4+ T cells and (B) CD8+ T cells (n=3/3). (C) Weight of the mice after 10 weeks of high cholesterol diet (n=11/10). (D) Spleen weight as percentage of body weight after 10 weeks of high cholesterol diet (n=10/8). (E) Cholesterol levels of CBL-Bfl/fl (n=20) and Cbl-bcKO (n=19) mice after 10 weeks of high cholesterol diet. (F) Atherosclerotic plaque volume in the aortic root of CBL-Bfl/fl (n=20) and Cbl-bcKO mice (n=19). (G) Quantification of necrotic core area in plaques of the aortic root (n=20/19). (H) Immunohistochemical quantification of plaque smooth muscle cell content (αSMA+, n=20/18) in the aortic root. (I) Histochemical quantification of collagen content (Sirius Red, n=19/17). (J) Immunohistochemical quantification of plaque macrophage (MAC3+, n=20/17) content in the aortic root. (K) Quantification of the number of CD3+ (n=20/17) T cells in the aortic root. Data is shown as mean ± SD, outliers were removed by ROUT test (Q = 1%) and normality was tested Shapiro-Wilk normality test. Normally distributed data was analysed by an unpaired 2-tailed student t-test and non-normally distributed data was analysed by Mann-Whitney U test. Statistical significance is displayed as * p < 0.05, ** p < 0.01, *** p < 0.001, not significant (ns). [file Image_1.tif]

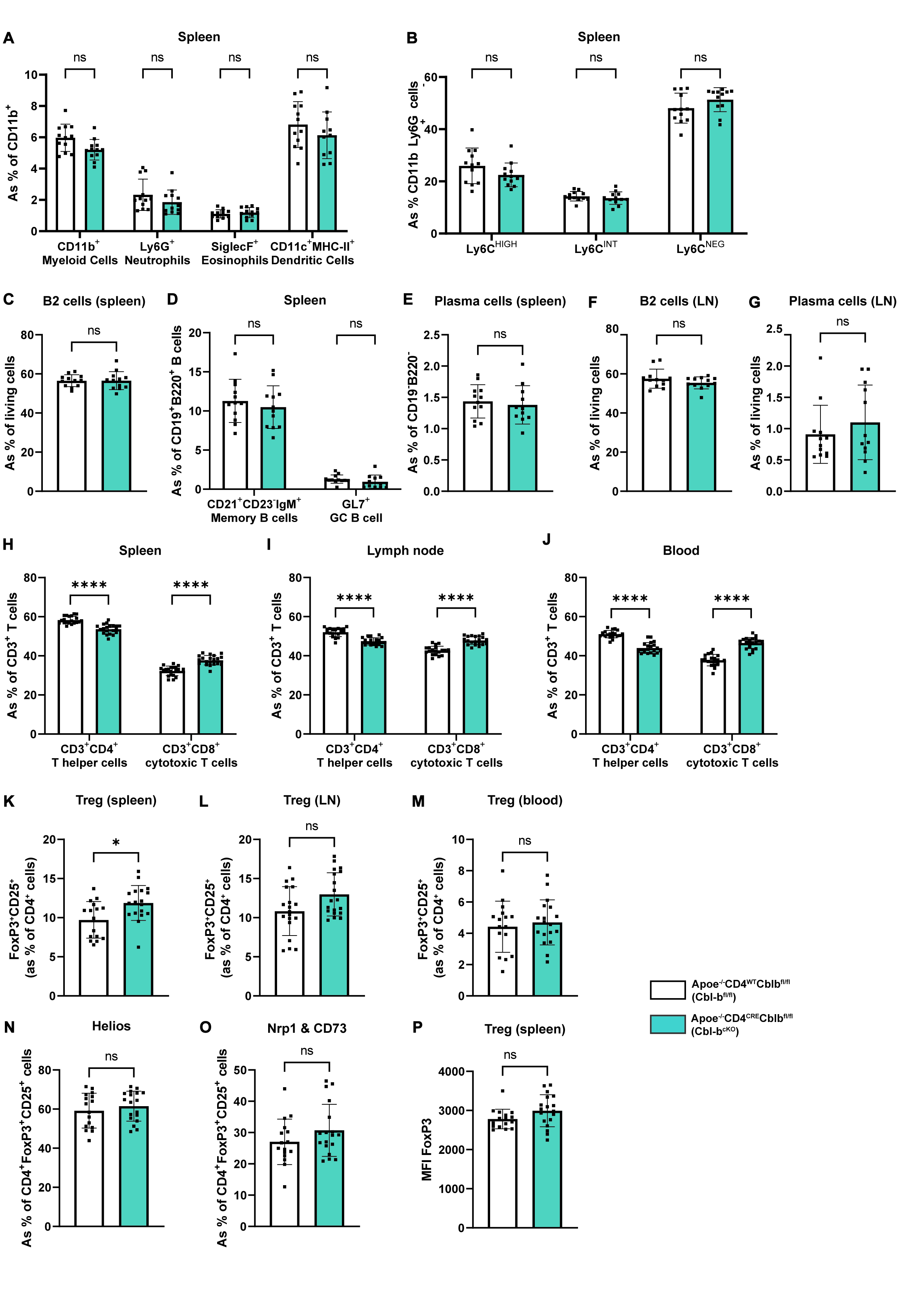

Supplement: Supplementary Figure 2 — (A) Percentage of CD11b+ myeloid cells (n=12/12), Ly6G+ neutrophils (n=12/12), siglecF+ eosinophils (n=11/12), and CD11c+MHC-II+ dendritic cells (n=12/11) in the spleen. (B) Ly6C expression level of CD11b+Ly6G- cells in the circulation (n=12/12). (C) Percentage of CD19+B220+ B cells in the spleen. (D) Frequency of CD21+CD23-IgM+ memory B cells (n=12/12), and GL7+ germinal center (GC) B cells (n=12/11). (E) Frequency of CD138+IgM+ plasma cells (n=12/11). Frequency of (F) B2 cells (n=12/12) and (G) plasma cells (n=12/12) in the lymph node. Percentage of CD4+ and CD8+ T cells in (H) spleen (n=20/20), (I) lymph node (LN), and blood (J) (n=19/20). Percentage of Tregs cells in (K) spleen (n=16/18), (L) lymph node (LN) (n=19/19), and (M) blood (n=16/18). Expression of (N) Helios and (O) double expression of Nrp1 and CD73, and (P) FoxP3 mean fluorescence intensity (MFI) in Tregs cells in the spleen (n=16/18). Data is shown as mean ± SD, outliers were removed by ROUT test (Q = 1%) and normality was tested Shapiro-Wilk normality test. Normally distributed data was analysed by an unpaired 2-tailed student t-test and non-normally distributed data was analysed by Mann-Whitney U test. Statistical significance is displayed as * p < 0.05, **** p < 0.0001., not significant (ns) [file Image_2.tif]

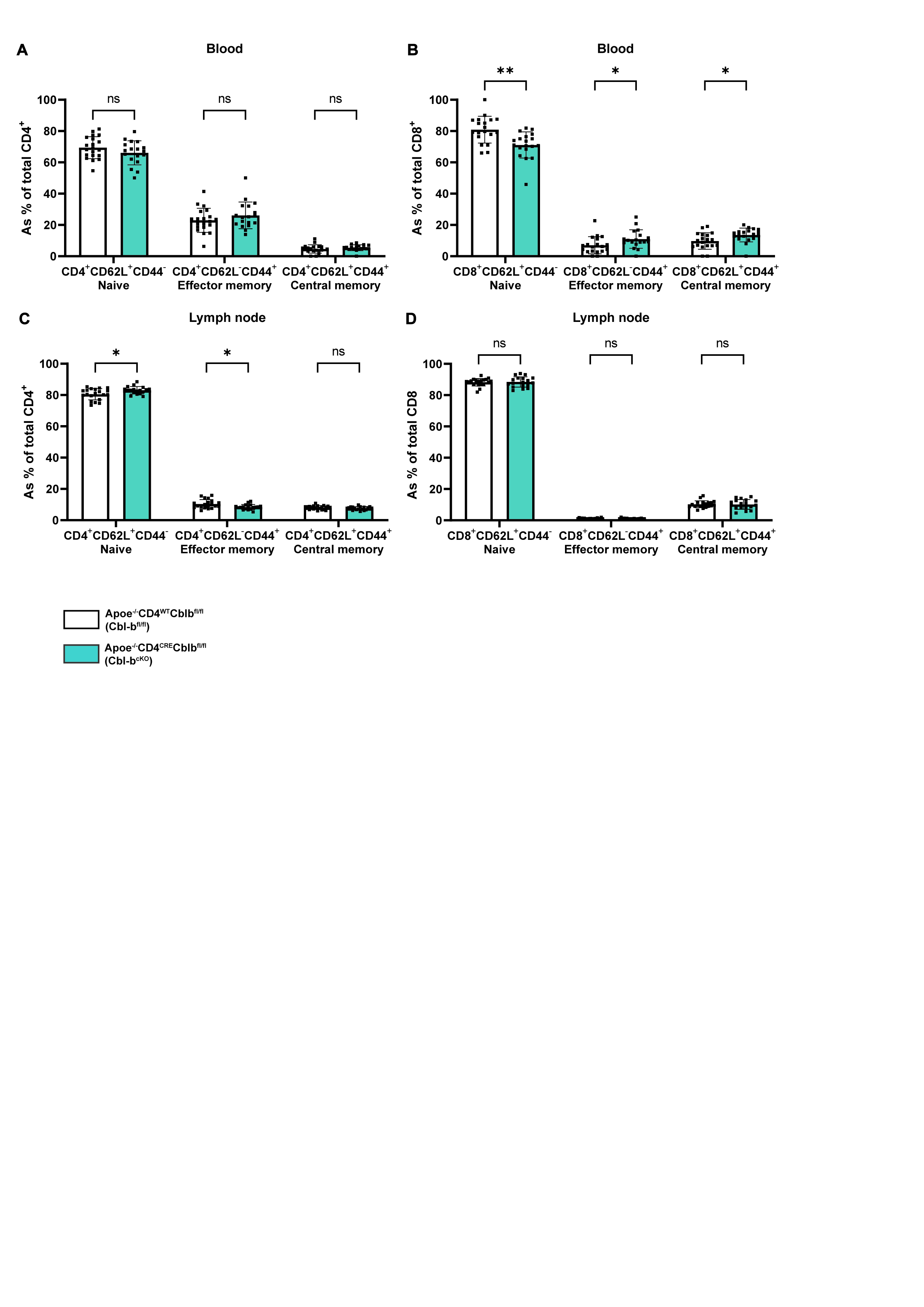

Supplement: Supplementary Figure 3 — Percentage of naive, effector, and central memory in the (A) blood and (C) lymph node CD4+ T cell population (n=20/18) and the (B) blood (n=19/19) and (D) lymph node (n=20/18) CD8+ T cell population. Data is shown as mean ± SD, outliers were removed by ROUT test (Q = 1%) and normality was tested Shapiro-Wilk normality test. Normally distributed data was analysed by an unpaired 2-tailed student t-test and non-normally distributed data was analysed by Mann-Whitney U test. Statistical significance is displayed as * p < 0.05, ** p < 0.01, not significant (ns). [file Image_3.tif]

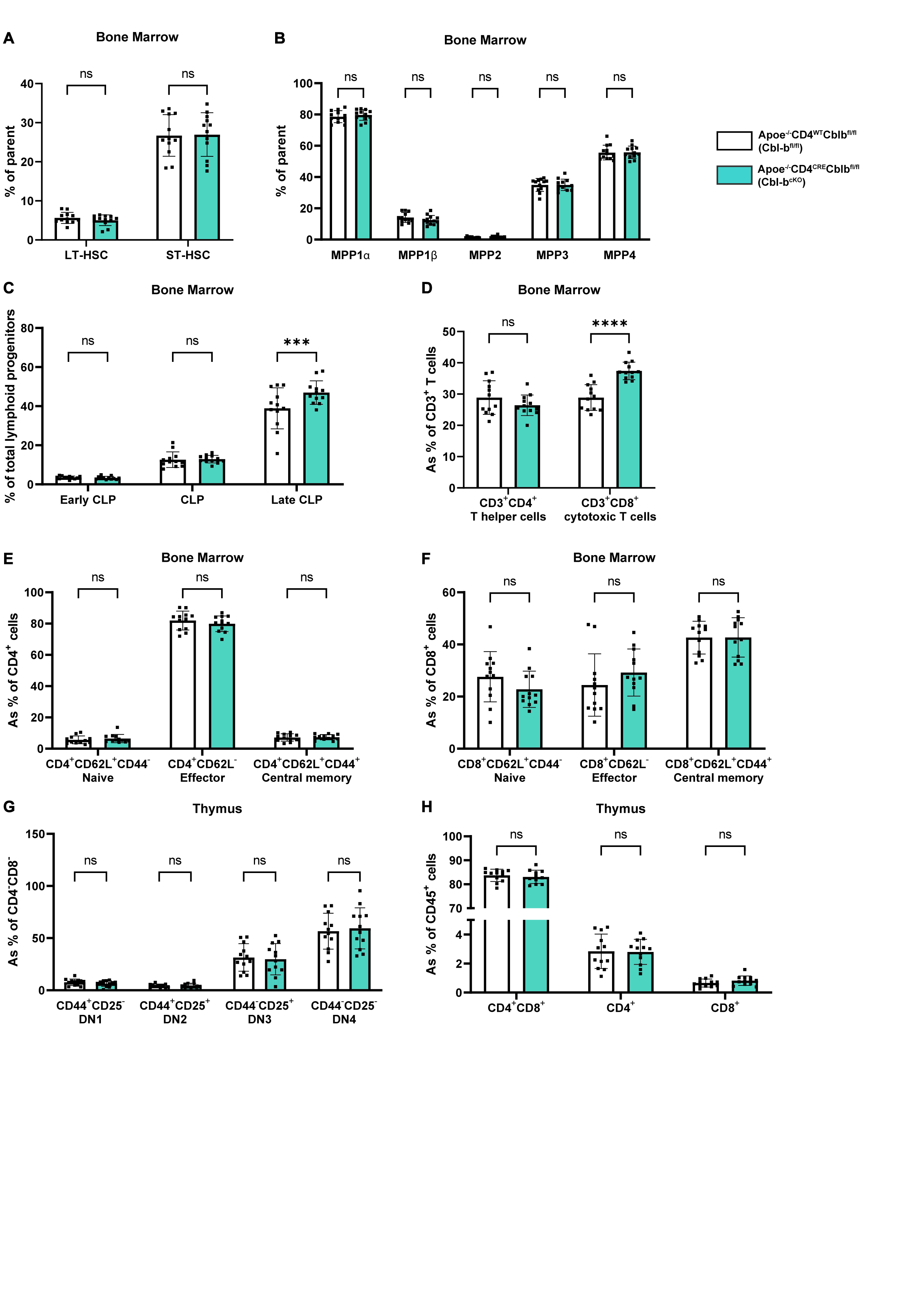

Supplement: Supplementary Figure 4 — (A–C) Flow cytometric analysis of stem cell population in the bone marrow (n=12/12). (D) Frequency of CD4+ and CD8+ T cells that have returned in the bone marrow (n=12/12). Percentage of naive, effector, and central memory in the (E) CD4+ T cell population and the (F) CD8+ T cell population in the bone marrow (n=12/12). (G, H) Developmental stages of T cells in the thymus (n=12/12). Statistical significance is displayed as *** p < 0.001, **** p < 0.0001, not significant (ns). [file Image_4.tif]
